# Development of a recombinase polymerase amplification (RPA) fluorescence assay for the detection of *Schistosoma haematobium*

**DOI:** 10.1186/s13071-019-3755-6

**Published:** 2019-11-04

**Authors:** Penelope Rostron, Tom Pennance, Faki Bakar, David Rollinson, Stefanie Knopp, Fiona Allan, Fatma Kabole, Said M. Ali, Shaali M. Ame, Bonnie L. Webster

**Affiliations:** 1 Faculty of Infectious and Tropical Diseases, London School of Hygiene and Tropical Medicine, London, UK; 2 Department of Life Sciences, Natural History Museum, London, UK; 3 London Centre for Neglected Tropical Disease Research (LCNTDR), London, UK; 4 Cardiff University, Cardiff, UK; 5 Public Health Laboratory - Ivo de Carneri, P.O. Box 122, Chake-Chake, Pemba United Republic of Tanzania; 6 Swiss Tropical and Public Health Institute, Socinstrasse 57, 4002 Basel, Switzerland; 7 University of Basel, Petersplatz 1, 4003 Basel, Switzerland; 8 Zanzibar Neglected Diseases Programme, Ministry of Health, P.O. Box 236, Zanzibar Town, Unguja United Republic of Tanzania

**Keywords:** *Schistosoma haematobium*, Urogenital schistosomiasis, Diagnostics, RPA, Isothermal, Molecular, Point-of-need (PON), Surveillance, Control, Elimination

## Abstract

**Background:**

Accurate diagnosis of urogenital schistosomiasis is vital for surveillance and control programmes. While a number of diagnostic techniques are available there is a need for simple, rapid and highly sensitive point-of-need (PON) tests in areas where infection prevalence and intensity are low. Recombinase Polymerase Amplification (RPA) is a sensitive isothermal molecular diagnostic technology that is rapid, portable and has been used at the PON for several pathogens.

**Results:**

A real time fluorescence RPA assay (RT-ShDra1-RPA) targeting the *Schistosoma haematobium* Dra1 genomic repeat region was developed and was able to detect 1 fg of *S. haematobium* gDNA. Results were obtained within 10 minutes using a small portable battery powered tube scanner device that incubated reactions at 40 °C, whilst detecting DNA amplification and fluorescence over time. The assay’s performance was evaluated using 20 urine samples, with varying *S. haematobium* egg counts, from school children from Pemba Island, Zanzibar Archipelago, Tanzania. Prior to RPA analysis, samples were prepared using a quick crude field DNA extraction method, the Speed Extract Kit (Qiagen, Manchester, UK). Positive assay results were obtained from urine samples with egg counts of 1–926 eggs/10 ml, except for two samples, which had inconclusive results. These two samples had egg counts of two and three eggs/10 ml of urine.

**Conclusions:**

The RT-ShDra1-RPA assay proved robust for *S. haematobium* gDNA detection and was able to amplify and detect *S. haematobium* DNA in urine samples from infected patients. The assay’s speed and portability, together with the use of crude sample preparation methods, could advance the rapid molecular diagnosis of urogenital schistosomiasis at the PON within endemic countries.

## Background

Schistosomiasis is a neglected tropical disease (NTD) caused by parasitic trematodes called schistosomes. *Schistosoma haematobium* is one of three main human-infecting schistosome species; with > 110 million cases of urogenital schistosomiasis, causing haematuria, bladder wall pathology, hydronephrosis leading to severe kidney disease [1, 2], and bladder cancer [3], with female and male genital schistosomiasis also being linked to infertility and HIV transmission [4]. It is the most commonly occurring schistosome species and is transmitted by various intermediate snail hosts of the genus *Bulinus* throughout Africa, parts of the Middle East, Madagascar and the Indian Ocean Islands [5] with a recent outbreak on the Mediterranean island of Corsica [6].

Sensitive and specific diagnostic tests are critical for the development, implementation and success of schistosomiasis control and elimination programmes [7–11]. They not only enable the accurate diagnosis and treatment of individual patients but also support the monitoring of control interventions [11–17]. Moreover, as a control programme achieves success, low infection intensity is common within the population, with a high proportion of those infected excreting low numbers of schistosome eggs that may escape detection by routine methods, namely urine filtration and haematuria detection strips [16, 17]. This increases the need for diagnostic sensitivity and specificity to prevent false negative diagnoses [7, 17]. The recently developed, and very promising, circulating anodic antigen (CAA) based test offers high sensitivity and is currently being optimized and evaluated for PON testing [18, 19].

Molecular diagnostics can be highly sensitive and specific [15]. Polymerase chain reaction (PCR) and quantitative PCR (qPCR) methods, that target and amplify schistosome DNA from urine and stool samples, have been shown to be sensitive (0.01–10 fg) and specific [20–23]. However, these methods are costly, take time, require a significant laboratory infrastructure and training, hampering their current use in endemic field settings [12, 15, 24]. Loop-mediated isothermal amplification (LAMP), overcomes some of these obstacles and has been successfully used in the field to diagnose human *S. haematobium* infections [25, 26].

Recombinase polymerase amplification (RPA) is an isothermal DNA amplification technology that can be performed in the field due to its low resource requirements. Reactions are rapid and take place at a low constant temperature using small portable devices and lyophilized reagents. DNA amplification can be detected either by gel electrophoresis, oligo chromatographic lateral flow (LF) strips or real time fluorescence, offering detection flexibility of use in endemic field settings [27–30]. A lateral flow RPA assay targeting the Dra1 repeat region (Dra1 LF-RPA) has already been developed for *S. haematobium* [31], and its specificity tested against other urinary pathogens. Here, we advance this research with the first laboratory development and testing of a novel fluorescence real time (RT) Dra1-RPA assay for *S. haematobium* (RT-ShDra1-RPA).

## Methods

### *Schistosoma haematobium* template DNA

For the assay development *S. haematobium* adult worm genomic DNA (gDNA) originating from the Zanzibar island Unguja was provided by the Schistosomiasis Collection at the Natural History Museum (SCAN) [32]. DNA was quantified using a Qubit 2.0 Fluorimeter (Invitrogen, California, USA) and diluted to a working concentration of 1 ng/µl in ddH_2_0.

### RPA assay development

#### RT-ShDra1-RPA primer design

**Fig. 1:**
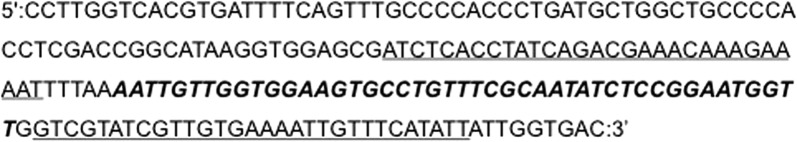
Dra1 repeat sequence showing the position of the RT-ShDra1-RPA primers (underlined) and probe (bold)

The Dra1 lateral flow RPA primers and internal probe (Dra1 LF-RPA) designed by Rosser et al. [31] were further adapted for RT-ShDra1-RPA amplification and detection. The internal probe was modified for the RT fluorescence-based detection as shown in Table 1 and Fig 1. RPA reactions were performed using the TwistAmp exo kit (TwistDX, UK) in 50 µl reactions containing 29.5 μl rehydration buffer, 2.1 μl of each of the forward and the reverse primers (10 pmol), 0.6 μl of the internal lateral flow probe (10 pmol), 12.2 μl of ddH_2_o and the DNA template (1 ng), which were added to the lyophilized RPA pellet. The reactions were initiated by the addition of 2.5 μl (280 mM) of magnesium acetate. The reactions were run at 40 °C for 20 minutes in an Axxin T-16 isothermal device (T-16 ISO) (http://www.axxin.com/Molecular-T16), which measures the increase in fluorescence, due to DNA amplification, over time. The reactions were manually mixed after 4 minutes.

**Table 1:** Sequences of the RT-ShDra1-RPA primers (forward and reverse) and the internal probe together with a description of the specific probe design for the assay

Primer/probe	Sequence (5′–3′)
Forward	ATCTCACCTATCAGACGAAACAAAGAAAAT
Reverse	AATATGAAACAATTTTCACAACGATACGAC
Probe	AATTGTTGGTGGAAGTGCCTGTTTCGCAA(FAM)(THF)(Q)CTCCGGAATGGTTG(C3) (46–52 bp long, 30 bp between 5′-end and THF with a minimum of 15 bp between the THF and the 3′-end of the probe; THF, a basic tetrahydrofuran residue or dSpacer (replaces any bp between the FAM and Q; C3, spacer at the 3’-end; Q, Quencher replaces a T; FAM, replaces a T)

#### RT-ShDra1-RPA sensitivity testing

Sensitivity was determined by running the RPA assay, as described above, using dilutions (1 ng, 1 pg, 1 fg and 0.5 fg) of the *S. haematobium* gDNA. Negative (no template) and positive (gDNA 1 ng) controls were run with the reactions.

### Pilot urine testing

As part of the Zanzibar Elimination of Schistosomiasis Transmission (ZEST) project (2011–2017) [33], 1.5 ml aliquots of urine samples from schoolchildren that participated in the annual surveys, were frozen at −20 °C, and kept at the Public Health Laboratory-Ivo de Carneri (PHL-IdC) on Pemba Island, Zanzibar. All urine samples included in the study presented here were collected in 2013 and were positive for *S. haematobium* eggs, identified by urine filtration (10 ml) during the ZEST parasitological surveys [33]. Twenty egg-positive urine samples were selected with a range of egg counts classified as very low, low, medium and high (Table 2). The samples were randomly selected from multiple Shehias, so as not to introduce any geographical biases, but they were stratified by egg count.

**Table 2:** Egg counts (per 10 ml of urine) for the urine samples tested, their egg count category (high > 400; medium 51–400; and very low 1–10) and their RPA results

Category	Egg count/10 ml	Urine code	RPA results
High	532	U1	+
Medium	102	U2	+
Very Low	8	U3	+
Medium	156	U4	+
High	816	U5	+
High	458	U6	+
Medium	145	U7	+
Medium	368	U8	+
High	750	U9	+
Medium	137	U10	+
High	742	U11	+
High	552	U12	+
High	926	U13	+
Medium	171	U14	+
Medium	68	U15	+
Very low	3	U16	+
Very low	1	U17	+
Very low	2	U18	?
Very low	3	U19	–
Very low	1	U20	+

*Key*: +, positive; –, negative; ?, cannot interpret

**Fig. 2:**
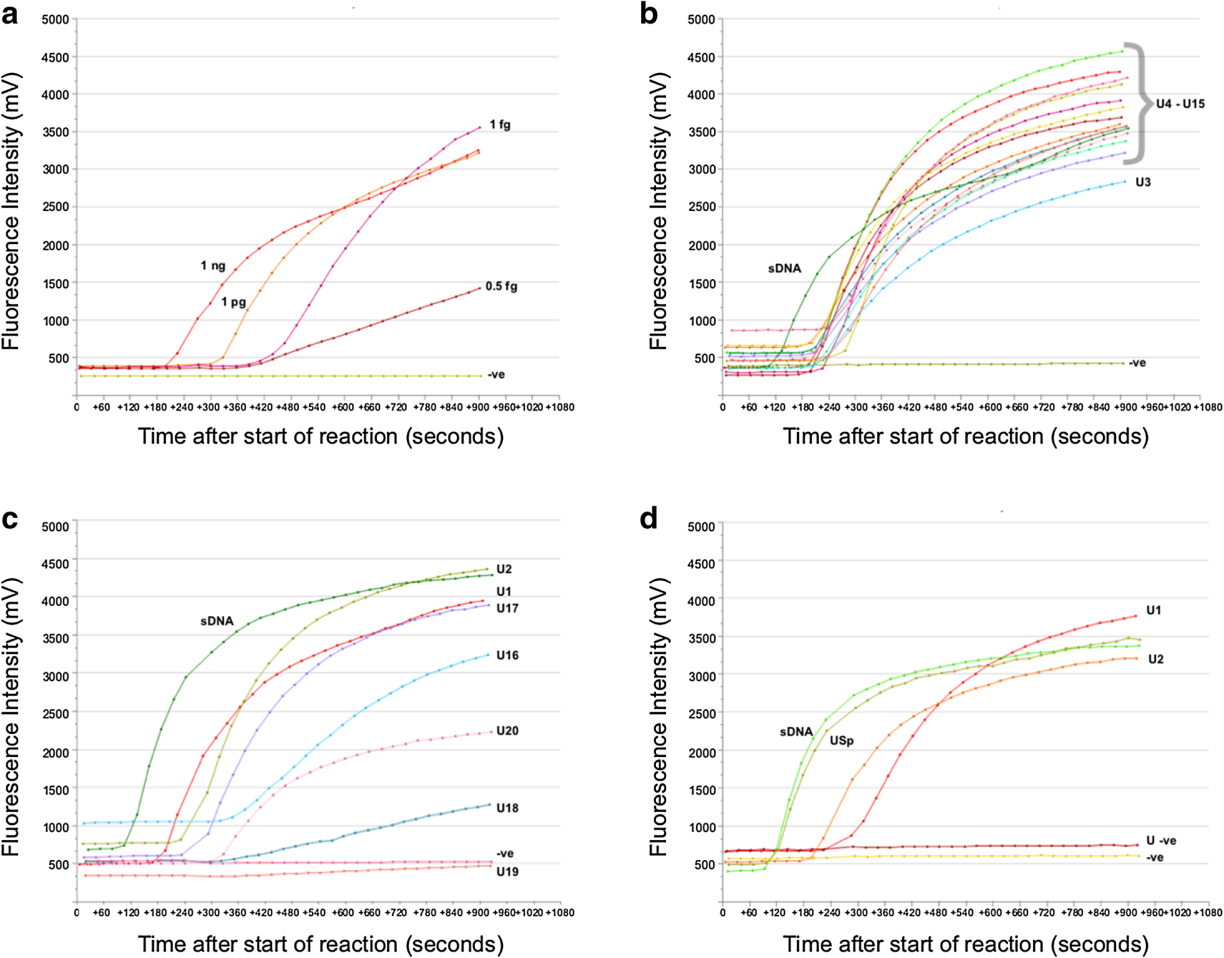
RPA fluorescent curves for the *S. haematobium* gDNA dilutions and the urine samples tested from PHL-IDC, Pemba. **a** *S. haematobium* gDNA dilutions. **b** Urine samples U3-15. **c** Urine samples U1+2 (high egg counts) and U16-20 (very low egg counts). **d** Urine samples U1+2 (high egg counts) and negative donor urine (U-ve) and negative donor urine spiked with *S. haematobium* gDNA (USp). sDNA corresponds to a DNA standard positive control. −ve corresponds to negative controls

*Notes*: There were no samples in the egg count range of 11–50. The urine code corresponds to the RPA curves shown in Fig. 2

At PHL-IdC, urine samples were defrosted, mixed and a 100 μl aliquot taken from each sample for DNA extraction. DNA was extracted, in country at PHL, from individual samples using the Qiagen Speed Extract kit (Qiagen). This is a fast, field-friendly and low-cost method for crude extraction of DNA using basic equipment. The protocol followed the manufacturerʼs recommendation with slight modification. All reagents were supplied within the Qiagen Speed Extract kit (Qiagen). 200 μl of EN buffer and 15 μl of the magnet bead mix was added to each urine sample, which was then mixed and incubated at room temperature for 3 min before being placed on a magnetic separation rack (New England Biolabs, Massachusetts, USA) for 1 min. During this time, DNA binds to the magnetic beads forming a pellet, allowing the supernatant to be removed and the pellet to be re-suspended in 100 μl of SL buffer to release the DNA from the beads. Samples were then heated at 95 °C for 5 min before returning to the magnetic rack for 1 min to pellet the magnetic beads. The supernatant, now containing extracted DNA, was removed and stored at room temperature. DNA samples were transported, at ambient temperature, to the Natural History Museum, London, UK, for RPA testing. Samples were analysed using the RT-ShDra1-RPA assay as described above using 5 μl of the DNA preparation in the 50 μl RPA reaction. A negative urine control (from a NHM laboratory staff member) and a positive control (donor urine spiked with *S. haematobium* gDNA) were also prepared using the Speed Extraction protocol and run alongside the urine samples from PHL-IdC.

## Results

### RT-ShDra1-RPA assay development and limit of detection

As shown in Fig. 2a, using 1ng of gDNA, the developed RT-ShDra1-RPA assay gave a positive fluorescent sigmoid curve which appeared after ~5 min of reaction time. There was a lower detection limit of 1 fg of gDNA with the final results obtained within 10 min.

### Urine sample examination

Fourteen urine samples were analysed from the medium and high egg count categories (Table 2), all of which gave a strong positive sigmoid fluorescent curve obtained within 8 min (Fig. 2b, c). As indicated in Table 2, for the six urine samples in the very low egg count category (1–10 eggs per 10 ml), the results were less easy to interpret. Four of the six samples produced strong positive sigmoid curves but one (U18, 2 eggs/10 ml) showed an inconclusive result (Fig. 2b, c) and one (U19, 3 eggs/10 ml) did not show any significant rise in fluorescence and was therefore considered negative. Positive and negative results were obtained from the spiked (USp) and non-spiked (U-ve) donor urine samples, respectively (Fig. 2d).

## Discussion

Schistosomiasis control programs aim to reduce disease morbidity and significant impact has been observed in certain areas, including the Zanzibar islands [8, 34–37]. However, progress in control and eventual elimination, of schistosomiasis, needs high performance diagnostic tests that can detect low levels of infection [7, 17]. The molecular detection of *S. haematobium* DNA in urine has been shown to be a highly sensitive indicator of infection [15, 22, 23]. RPA is a DNA amplification and detection technology that is particularly suitable for PON use [24, 31–37], as all reagents are readily available lyophilized with the main RPA reagents provided in a single dried pellet, simplifying assay preparation, reducing contamination and allowing easy transportation and long-term storage at room temperature. Additionally, DNA amplification occurs at low constant temperatures between 25–40 °C and real time fluorescence amplification detection can be performed using small portable tube-scanner devices, with results obtained within 20 minutes.

Here, a real time fluorescence-based RPA assay was successfully developed to amplify the Dra1 DNA repeat region of *S. haematobium* (RT-ShDra1-RPA). The present RT-ShDra1-RPA lower limit of detection was 1 fg of *S. haematobium* gDNA, lower than the existing Dra1 end point PCR diagnostic assay, that has a detection limit of 10 fg of *S. haematobium* gDNA [38], and also lower than the detection limit of the developed lateral flow RPA Dra1 assay (LF-ShDra1-RPA) [31], that had a detection limit of 100 fg of gDNA. However, the Dra1 qPCR and LAMP assays have been reported to display higher sensitivities detecting as low as 1 fg and 0.1 fg of *S. haematobium* gDNA, respectively [15, 20–23, 25, 26]. Theoretically, RPA can detect a single copy of a DNA target thus there is scope for further assay optimization and development, particularly focusing upon different target regions, probe and primer combinations and concentrations to increase assay sensitivity.

RPA has been shown to offer degrees of tolerance to inhibitors found in urine, and works well on crudely prepared samples [30] reducing the need for the resources needed for sophisticated sample preparation and purification methods, further enhancing its feasibility for PON use. The RT-ShDra1-RPA developed here gave positive results when tested on crudely prepared urine samples from infected school children from the endemic Pemba Island, Zanzibar. The DNA preparations took ~15 minutes for 12 samples and required only portable low powered equipment. The assay produced strong positive results for samples that had medium to high egg counts (11–926 eggs per 10 ml), performing as well as microscopy but further analysis of samples with lower egg counts is warranted, particularly as this is the main application for this type of diagnostic. Results varied between samples that had between 1–10 eggs per 10 ml. Four among the 6 samples, within this egg count range, gave positive results but one was inconclusive and one was negative. This maybe due to there not being eggs present in the aliquot of urine analysed, false positive egg identification by microscopy and/or sample degradation. It is worth mentioning that two of the three urine filtration reads (each sample was read by three technicians) performed on these samples were recorded as egg-negative. Further testing of low egg count samples and egg-negative samples is now needed to further assess the performance of the developed assay. It is also necessary to determine if this method is detecting DNA from the *S. haematobium* eggs or Cell-Free-Parasite DNA (CFPD), which has been reported as a source of DNA in PCR and qPCR assays [15, 39]. RPA reactions are only semi-quantitative with the time to onset of amplification being longer for low and quicker for high DNA concentrations (Fig. 2a) and hence, could currently be used preferentially to identify infected individuals, but not to evaluate infection intensities.

The extraction method used for this study was chosen due to its field applicability and in particular its low resource needs, speed and simplicity. The SpeedXtract method works well for RPA assays as high quality DNA and purity is not needed. However, comparisons with other standard extraction methods would be beneficial as this may increase sensitivity. Particularly, as laboratories in endemic countries grow their infrastructure, more high resource sample preparation methods may become feasible.

Although the ShDra1-RPA assayʼs primer and probe combinations have proved negative with regard to their cross-reactivity with other pathogens found in urine samples and also other schistosome species, this has only been fully tested in the lateral flow form [31]. Here the developed RT-ShDra1-RPA assay is predicted to show the same specificity as the primer and probes used are identical except that they are modified for fluorescent detection. However, full validation on reference pathogen samples and also testing on a range of well-defined negative clinical samples is warranted to further validate the RT-ShDra1-RPA assayʼs clinical specificity.

Defining the gold standard for the diagnosis of schistosomiasis is not easy and there is no real current consensus. Currently egg detection in clinical samples is commonly used but this is known to not have the clinical sensitivity needed in low prevalence and intensity settings. Diagnosis by qPCR [20–23] is also used as a gold standard in some laboratories with CAA testing also coming on to the agenda however, comparisons between all tests are warranted. In this study the RT-ShDra1-RPA assay has been tested with regard to egg count in a small set of samples but the assay certainly needs testing on a wider range, and on a number of well-defined samples together with direct comparisons to qPCR and CAA [18, 19]. These comparisons are needed to define the diagnostic use case, for such an assay as RPA, in relation to its possible utility at different stages of schistosomiasis control [10–13]. Certainly, the characteristics of RPA could support diagnosis and possible test and treat scenarios at the elimination stage.

## Conclusions

The developed RT-ShDra1-RPA assay offers an alternative *S. haematobium* DNA amplification system that has the potential use as a molecular diagnostic tool for urogenital schistosomiasis in endemic settings. Results can be rapidly obtained from crudely prepared samples using small portable devices requiring minimal infrastructure. Further development is needed to test its sensitivity and performance in low prevalence settings, where infection intensities and egg counts are mostly low, and in comparison to existing diagnostic tests.

## Data Availability

All data involved and arising from the study are included in the publication. Samples are available upon request.

## Abbreviations

RPA: recombinase polymerase amplification
PCR: polymerase chain reaction
qPCR: quantitative polymerase chain reaction
Sh: *S. haematobium*
CFPD: cell-free parasite DNA
gDNA: genomic DNA
U: urine
sDNA: synthetic DNA standard
-ve: negative control
